# Fine-Tuned Large Language Model for Extracting Patients on Pretreatment for Lung Cancer from a Picture Archiving and Communication System Based on Radiological Reports

**DOI:** 10.1007/s10278-024-01186-8

**Published:** 2024-07-02

**Authors:** Koichiro Yasaka, Jun Kanzawa, Noriko Kanemaru, Saori Koshino, Osamu Abe

**Affiliations:** Department of Radiology, Graduate School of Medicine, The University of Tokyo, 7-3-1 Hongo, Bunkyo-ku, Tokyo, 113-8655 Japan

**Keywords:** Lung neoplasms, Radiology information systems, Deep learning

## Abstract

This study aimed to investigate the performance of a fine-tuned large language model (LLM) in extracting patients on pretreatment for lung cancer from picture archiving and communication systems (PACS) and comparing it with that of radiologists. Patients whose radiological reports contained the term lung cancer (3111 for training, 124 for validation, and 288 for test) were included in this retrospective study. Based on clinical indication and diagnosis sections of the radiological report (used as input data), they were classified into four groups (used as reference data): group 0 (no lung cancer), group 1 (pretreatment lung cancer present), group 2 (after treatment for lung cancer), and group 3 (planning radiation therapy). Using the training and validation datasets, fine-tuning of the pretrained LLM was conducted ten times. Due to group imbalance, group 2 data were undersampled in the training. The performance of the best-performing model in the validation dataset was assessed in the independent test dataset. For testing purposes, two other radiologists (readers 1 and 2) were also involved in classifying radiological reports. The overall accuracy of the fine-tuned LLM, reader 1, and reader 2 was 0.983, 0.969, and 0.969, respectively. The sensitivity for differentiating group 0/1/2/3 by LLM, reader 1, and reader 2 was 1.000/0.948/0.991/1.000, 0.750/0.879/0.996/1.000, and 1.000/0.931/0.978/1.000, respectively. The time required for classification by LLM, reader 1, and reader 2 was 46s/2539s/1538s, respectively. Fine-tuned LLM effectively extracted patients on pretreatment for lung cancer from PACS with comparable performance to radiologists in a shorter time.

## Introduction

Lung cancer is the leading cause of cancer-related death worldwide [1], with its incidence reported as 0.45–0.86% during lung cancer screening with low-dose computed tomography (CT) [2]. Researchers have endeavored to improve detection and diagnostic performance by incorporating radiomics [3], machine learning [4], and deep learning technologies [5]. Despite their merits, applications of these advanced technologies necessitate a large-scale dataset to ensure optimal performance [6, 7]. However, collecting datasets is not a simple task because of the ambiguity and complexity of natural language. Searching picture archiving and communication systems (PACS) for patients with lung cancer using the term “lung cancer” results in the inclusion of those with posttreatment status. Ineligible patients should be excluded. However, there are several terms that indicate status after treatment, for example, after chemotherapy, treated with cisplatin, treated with immune checkpoint inhibitor, and after chemoradiation therapy. Thus, sorting patient status after treatment is an arduous endeavor.

Most recently, ChatGPT with OpenAI user interface (hereafter denoted as ChatGPT), a chatbot system based on a generative pretrained transformer, has gained widespread interest. Despite its excellent performance in the natural language tasks [8], this algorithm requires data to be uploaded to the server via the internet. Therefore, protection of privacy must be ensured during this process [9]. This hinders the application of ChatGPT to large-scale data. Conversely, some other pretrained large language models (LLMs), which can be classified into encoder only, decoder only, and encoder-decoder types, can be downloaded to local computers. For classification tasks, encoder only LLMs (such as Bidirectional Encoder Representations from Transformers) are typically used. Although such pretrained LLMs are not specifically trained for medical purposes, they can be fine-tuned using data from the field of radiology to derive optimized output. Such fine-tuned LLMs can potentially extract eligible patients from PACS with high performance in a short duration, which would result in facilitating future large-scale studies. In addition, automated classification system for the pretreatment lung cancer can be used as an alerting system, which would become a fail-safe solution for radiologists. However, to our knowledge, there have been no studies regarding fine-tuning of NLP models to classify patients on pretreatment for lung cancer based on radiological reports in any language.

This study aimed to investigate the performance of fine-tuned LLM for Japanese language in extracting patients on pretreatment for lung cancer in CT reports from PACS compared with that of radiologists.

## Materials and Methods

Our Institutional Review Board approved this retrospective study, which waived the requirement for obtaining written informed consent from patients because of the retrospective nature of this study.

### Patients

**Fig. 1:**
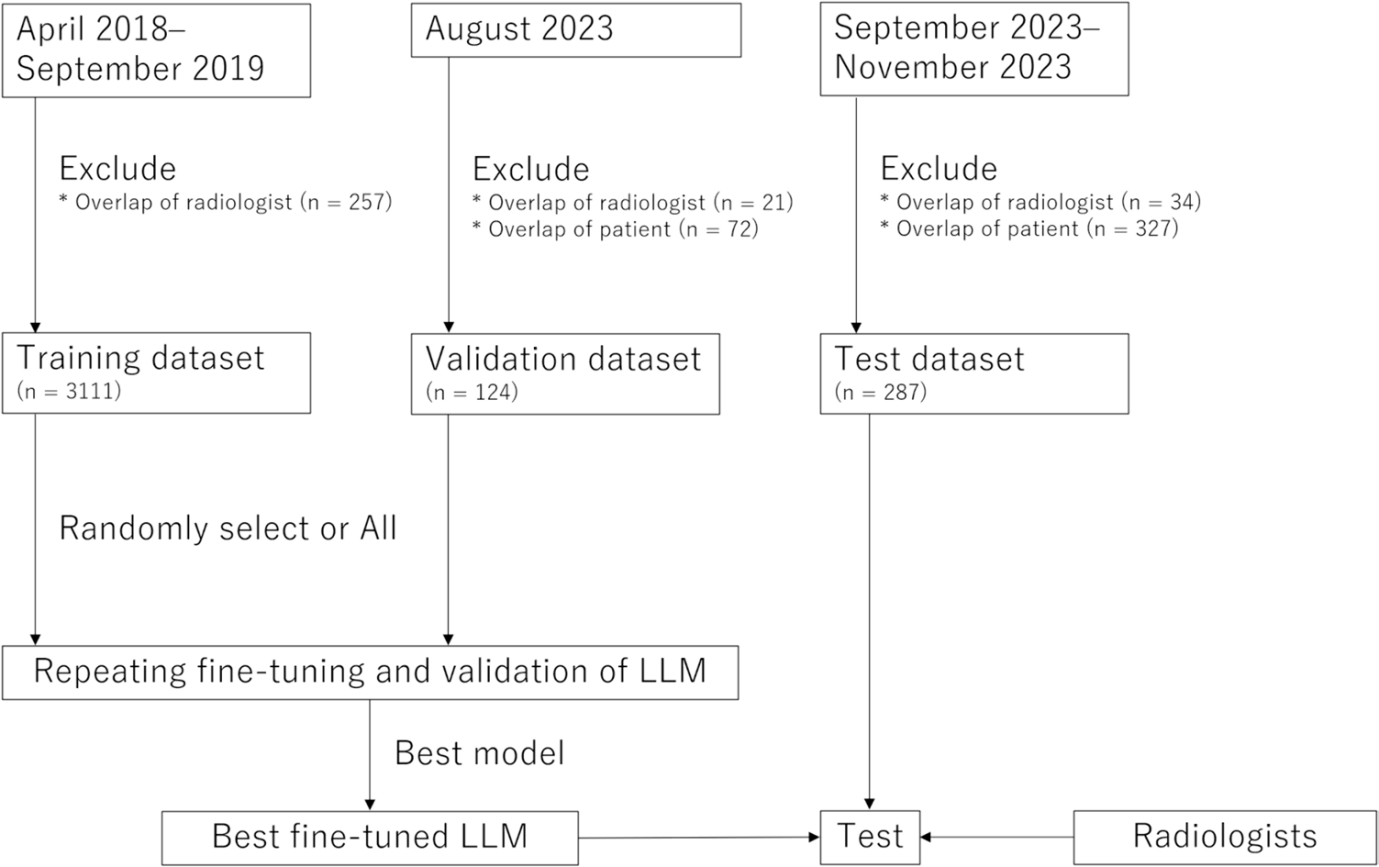
Patient inclusion and exclusion process. LLM, large language model

A radiologist (radiologist A) with a 13-year imaging experience searched PACS for patients whose CT radiological report included the term lung cancer. Patients who underwent CT from April 2018 to September 2019, August 2023, and September 2023 to November 2023 were included in the training, validation, and test datasets, respectively. To avoid overlap of radiologists who confirmed the radiological report for the clinical purpose between training vs. validation and test datasets, 257, 21, and 34 were excluded from the training, validation, and test datasets, respectively. This was performed in order to enhance the generalizability of the study results (i.e., to validate or test the trained model’s performance on radiological reports confirmed by different radiologists from the training dataset). As a result, through this process, reports confirmed by radiologists with a 16-year imaging experience or more, whose working period at our institution spanned over the time interval between training and validation/test, were excluded from the validation and test datasets. Additionally, to avoid overlap of patients between the training vs. validation and test datasets, 72 and 327 patients were excluded from the validation and test datasets, respectively. Hence, 3111, 124, and 287 patients were included in the training, validation, and test datasets, respectively (Fig. 1). Their radiological reports, including the clinical indication section, imaging diagnosis section, and patient background information, were retrieved in CSV format. All radiological reports were written in Japanese with free text style and were confirmed by radiologists with imaging experience of 5 or more years.

### Reference Standard

The clinical indication section and imaging diagnosis section of the radiological report were reviewed, and the report was classified into four groups: group 0, patient without lung cancer; group 1, patient with pretreatment lung cancer (excluding CT planning for radiation therapy); group 2, patient after lung cancer treatment; and group 3, CT examination performed for the radiation therapy planning. For the training and validation datasets, these evaluations were performed by radiologist A. For the test dataset, radiologist A and another radiologist (radiologist B with a 6-year imaging experience) created the reference standard with consensus reading, because the results in this dataset are the key data for this study.

### Fine-Tuning of the Pretrained LLM

**Fig. 2:**
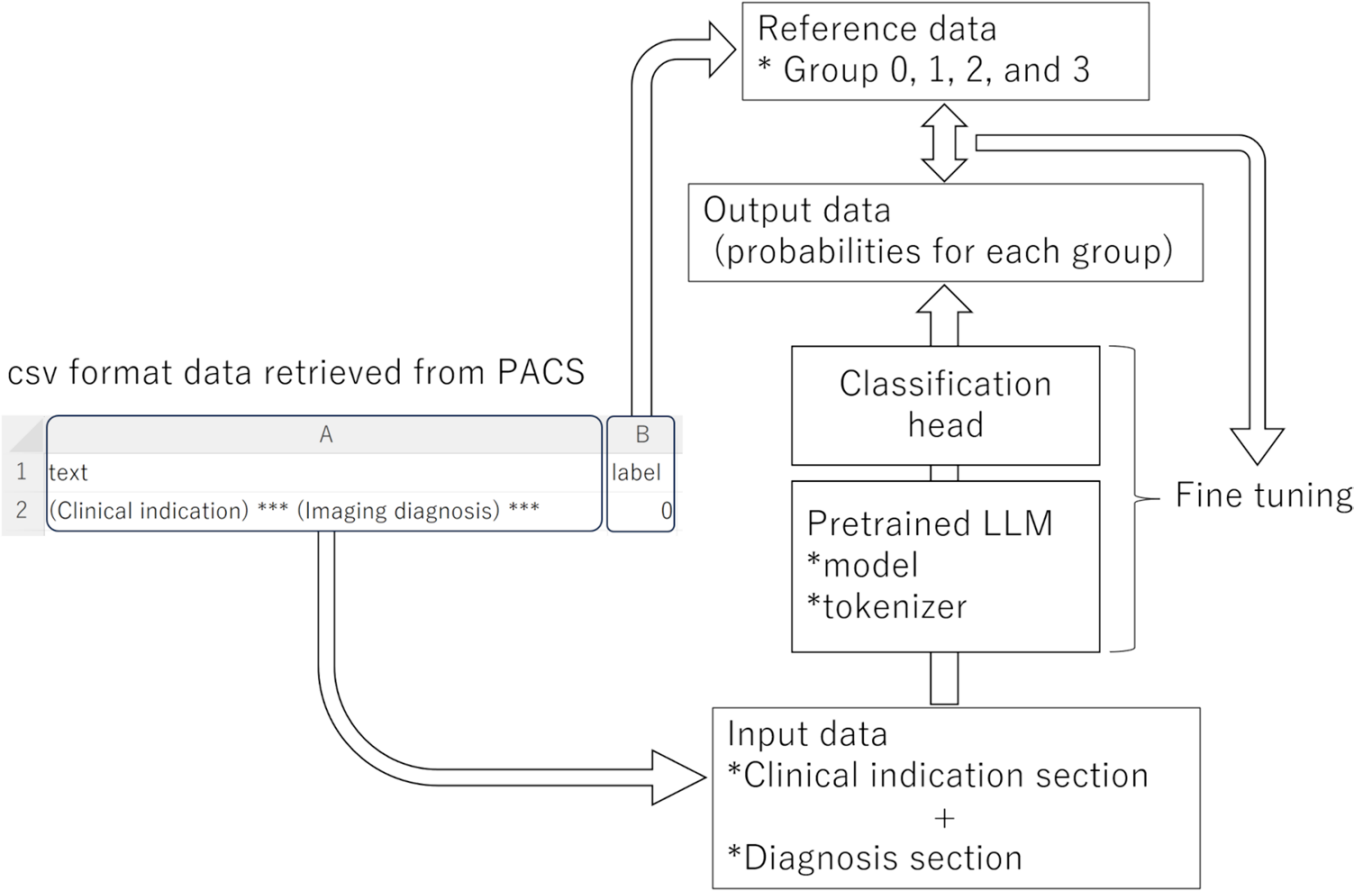
Algorithm of fine-tuning LLM for report classification. LLM, large language model; PACS, picture archiving and communication system

Fine-tuning of the pretrained Bidirectional Encoder Representations from Transformers Japanese model (https://huggingface.co/cl-tohoku/bert-base-japanese) was conducted using the programming language of Python version 3.10.13 (https://www.python.org/) and Transformers library version 4.35.2 (https://huggingface.co/) on a workstation equipped with a central processing unit (Core™ i9-12900F, Intel), a graphic processing unit (GeForce RTX 3090, NVIDIA), and a 128-GB RAM. The model, which consisted of 12 layers, 768 dimensions of hidden states, and 12 attention heads, had been pretrained with Japanese Wikipedia as of September 1, 2019. Using the AutoModelForSequenceClassification class method in the Transformers library, the model was set to classify reports, which consisted of the clinical indication and imaging diagnosis section, into four groups based on the logits for each group (Fig. 2). The number of epochs (set at 10) for the fine-tuning process was empirically determined using the training and validation datasets. Other hyperparameters were set to default values of the Transformers library. To evaluate the effect of the number of datasets on the performance, the fine-tuning and validation processes were repeated by changing the number of training datasets (sessions 1, 2, 3, and 4 with 10, 100, 1000, and 3111 patients, respectively). The performance of the non-fine-tuned BERT in the validation dataset was also assessed. To manage the imbalance in the number of patients across groups, undersampling of the group 2 data was performed (497 out of 2480 patients were randomly selected). The downsampling size for group 2 (497 out of 2480) was determined so that the size for this group becomes identical to that for group 1 (497). This dataset was also utilized in session 5. The required time for training and accuracy in the validation dataset was recorded. In each session, fine-tuning and validation were conducted 10 times. Then, the median of the required time and accuracy in each session were calculated. The code used for fine-tuning can be made available upon reasonable request.

### Test Phase of the Fine-Tuned LLM

The best-performing model in the validation dataset was further evaluated in the independent test dataset. Two other radiologists (readers 1 and 2 with 4- and 1-year imaging experience, respectively) were also involved in manually classifying the reports in the test dataset into four groups. The classified group data and the time required to complete the task were recorded.

### Statistical Analyses

Statistical analyses were performed using R version 4.1.2 (https://www.r-project.org/). For the comparisons of continuous and nominal variables in patient background, analysis of variance and chi-squared test were performed, respectively. Using the Wilcoxon signed rank test, the median accuracy of the LLM in the validation dataset was compared between sessions 4 and 1–3. The sensitivity for each group and overall accuracy in the test dataset were compared between the fine-tuned LLM *vs.* readers using the McNemar test. The diagnostic performance of the fine-tuned LLM in differentiating group 1 from other groups was evaluated by calculating the area under the receiver operating characteristic curve (AUC) based on the probability for group 1 calculated from logit data. The association between the years of imaging diagnosis of the radiologists who confirmed the report *vs.* the accuracy in the test dataset was evaluated using Spearman’s rank correlation coefficient. The inter-rater agreement between the two annotators in the test dataset was calculated with Cohen’s kappa analysis. Statistical significance was set at a *p* value of 0.050.

## Results

### Patients

Background information on the patients is provided in Table 1. The numbers of patients in group 0/1/2/3 were 37/497/2480/97, 2/20/98/4, and 4/58/224/1 for the training, validation, and test datasets, respectively. There was no statistically significant difference in age, sex, and the number of patients in each group between the training, validation, and test datasets. The years of imaging diagnosis of radiologists who confirmed the reports in the test dataset were 5–13 years.

**Table 1:** Patient background information

	Training	Validation	Test	Comparison
Number of patients	3111	124	287	
Age (years)	71.8 ± 10.0	72.3 ± 10.4	72.1 ± 10.5	0.760
Sex (male/female)	1945/1166	76/48	170/117	0.523
Number of radiologists who confirmed report	15	12	12	
Number of patients in each group				0.115
0: lung cancer absent	37	2	4	
1: Pretreatment lung cancer present	497	20	58	
2: After treatment for lung cancer	2480	98	224	
3: CT for planning radiation therapy	97	4	1	

Comparisons were performed with analysis of variance and chi-squared test for the continuous and nominal variables, respectively. *P*-values are provided in the “Comparison” column

*P*-values are provided in the “Comparison” column

### Association Between the Number of Training Datasets and Performance in the Validation Dataset

**Fig. 3:**
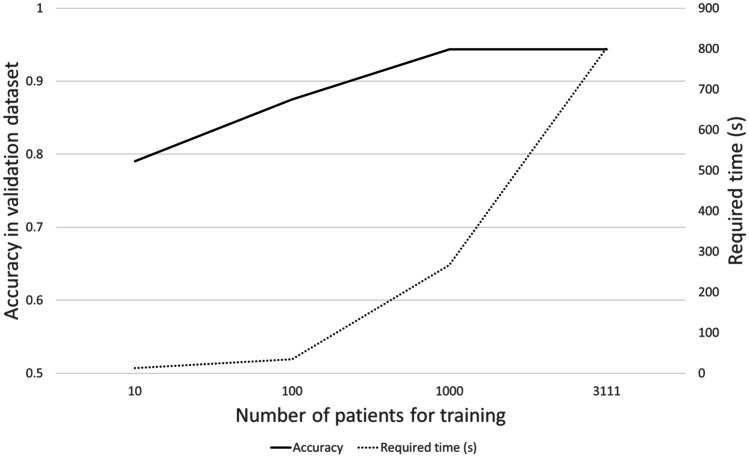
Relationship between the number of training dataset *vs.* accuracy in the validation dataset and required training time

The accuracy of the non-fine-tuned model was 0.056. By increasing the number of patients from 10, 100, 1000, to 3111, the median accuracy of the model changed from 0.790, 0.875, 0.944, to 0.944, respectively (Fig. 3). Statistically significant differences were found in the accuracy of the fine-tuned LLM between sessions 4 and sessions 1 and 2 (*p* = 0.005 and *p* = 0.006, respectively). No statistically significant difference was observed in the accuracy of LLM between sessions 4 and 3 (*p* = 1.000). The median time required for the training in sessions 1, 2, 3, and 4 were 13 s, 35 s, 267 s, and 803 s, respectively.

### Effect of Undersampling on the Sensitivity of Each Group in the Validation Dataset

As for the best performed model in session 4, the sensitivity for group 0/1/2/3 was 1.000/0.900/0.969/1.000. Because the relatively lower sensitivity in group 1 resulted from the relatively smaller number of patients included in this group (*n* = 497) compared with those in group 2 (*n* = 2480), an undersampling technique was employed to group 2 data by excluding randomly selected group 2 patients (session 5). The median accuracy and time required for the training in session 5 were 0.944 and 303s, respectively. As for the best performed model in session 5, the sensitivity for group 0/1/2/3 was 1.000/0.950/0.959/1.000, and improvement in the sensitivity for group 1 as compared to session 4 was noted. Because the sensitivity of group 1 is more important than that of group 2, the best model in session 5 was selected for further evaluation of its performance in the test dataset.

### Performance of the Fine-Tuned LLM and Radiologists in the Test Dataset

**Fig. 4:**
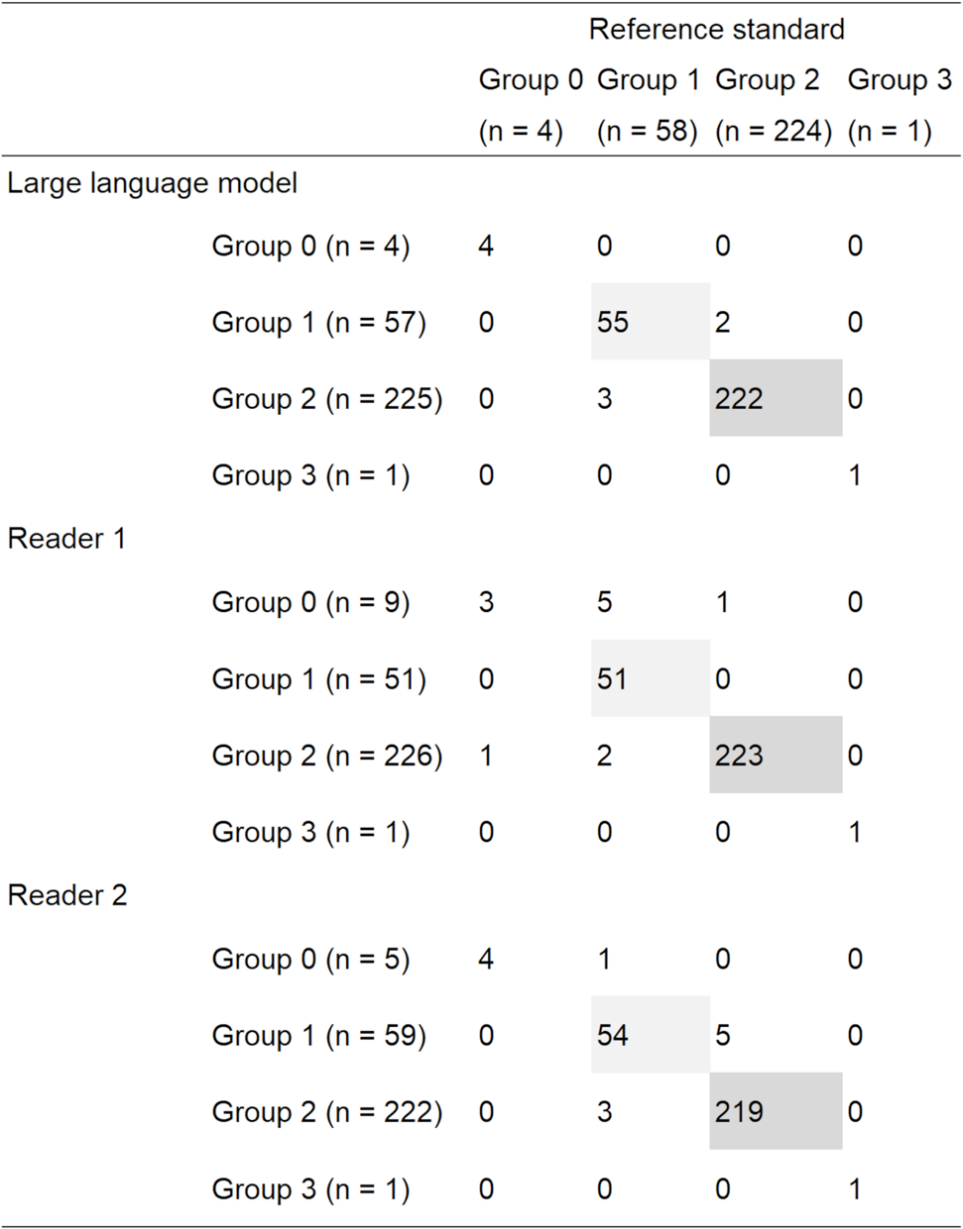
Confusion matrix for a reference standard vs. prediction data in the test dataset. Cells with high frequency data are highlighted with gray

Confusion matrix for the reference standard *vs.* prediction data by the best fine-tuned LLM and radiologists is provided in Fig. 4. In Table 2, the sensitivity and accuracy data are illustrated. The accuracy of the fine-tuned LLM (0.983) tended to be superior to that of readers 1 (0.969) and 2 (0.969). The sensitivity in differentiating group 1 for the fine-tuned LLM (0.948) tended to be superior to that for readers 1 (0.879) and 2 (0.931). The sensitivity in differentiating group 2 patients for the fine-tuned LLM was almost perfect (0.991) and comparable to that for readers 1 (0.996) and 2 (0.978). The fine-tuned LLM correctly distinguished group 0 and 3 patients with a sensitivity = 1.000 for both.

**Table 2:** Data for sensitivity and time required in the test dataset

	Fine-tuned LLM	Reader 1		Reader 2	
		Score	Comparison	Score	Comparison
Accuracy	0.983 (282/287)	0.969 (278/287)	0.343	0.969 (278/287)	0.343
Sensitivity for each group					
Group 0	1.000 (4/4)	0.750 (3/4)	1.000	1.000 (4/4)	1.000
Group 1	0.948 (55/58)	0.879 (51/58)	0.221	0.931 (54/58)	1.000
Group 2	0.991 (222/224)	0.996 (223/224)	1.000	0.978 (219/224)	0.371
Group 3	1.000 (1/1)	1.000 (1/1)	1.000	1.000 (1/1)	1.000
Time required (s)	46	2539		1538	

Comparisons between fine-tuned LLM *vs.* readers were performed using the McNemar test

*P*-values are provided in the “Comparison” columns

*LLM* large language model

The diagnostic performance for discriminating group 1 from other groups using output probability for this group was high with an AUC = 0.970 (95% confidence interval, 0.927–1.000).

The fine-tuned LLM classified all reports in the test dataset within 46 s in the absence of a graphic processing unit. This was 55.2- and 33.4-fold faster than that of readers 1 (2539 s) and 2 (1538 s), respectively.

The inter-rater agreement between the two annotators in the test dataset was 0.874.

### Association Between the Years of Imaging Diagnosis of the Radiologists Who Confirmed the Report vs. the Accuracy in the Test Dataset

**Fig. 5:**
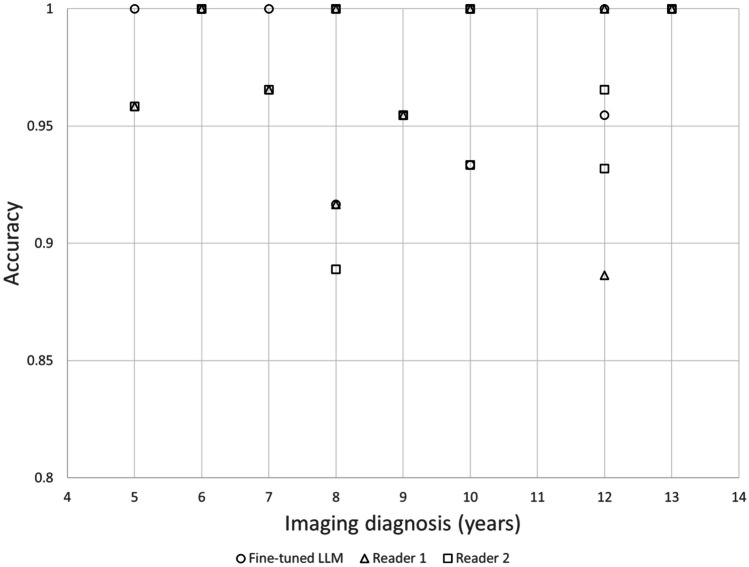
Association between years of the imaging diagnosis of radiologists who confirmed CT radiological reports *vs.* the accuracy in the test dataset. LLM, large language model

No significant correlation was found between the years of imaging diagnosis of the radiologists who confirmed the report *vs.* the accuracy in the test dataset of the fine-tuned LLM (*r* =  − 0.120, *p* = 0.696), reader 1 (*r* = 0.177, *p* = 0.564), and reader 2 (*r* = 0.025, *p* = 0.936) (Fig. 5).

## Discussion

Extracting patients on pretreatment for lung cancer from the PACS requires the exclusion of several ineligible patients, such as those after treatment; however, this process was an arduous task owing to the ambiguity and complexity of natural language. In this study, we found that a fine-tuned LLM effectively extracted patients on pretreatment for lung cancer with comparable performance to radiologists in a short time.

The overall accuracy in classifying reports into four groups was similar between the two readers (0.969). The fine-tuned LLM showed a slightly higher accuracy (0.983) compared with other methods. This suggests that the performance of the fine-tuned LLM was comparable to that of the radiologists. Among the four groups, the sensitivity for group 1 (pretreatment lung cancer present) would be the most relevant indicator. The diagnostic performance of the fine-tuned LLM in differentiating patients in this group was high, with an AUC of 0.970. The time required for inference by the fine-tuned LLM was 33.4–55.2-fold faster than that of the radiologists. Thus, our proposed method would facilitate the extraction of eligible patients in future studies, especially in large-scale studies, such as deep learning model development and machine learning model incorporating radiomics features. Additionally, our method may have another practical merit: building an alerting system. It is anticipated that radiologists would alert physicians when the imaging findings indicate an emergency or incidental malignant lesions were found. Our method may have the potential to serve as a fail-safe solution for radiologists.

Usefulness of the Bidirectional Encoder Representations from Transformers model for Chinese language in lung cancer radiological report was also demonstrated by Hu et al. [10]. Their developed model was found to effectively extract information about lung cancer staging from CT reports with macroaverage F1 score of 80.97, which was better than bidirectional long short-term memory networks-conditional random field (77.27). This would indicate the usefulness of Bidirectional Encoder Representations from Transformers model over the conventional natural language model.

Overfitting is a phenomenon when a model has become too attuned to the training dataset. Therefore, it is desirable to evaluate the model’s performance on datasets which were not used in the training process. For the validation and test datasets, we used radiological reports which were confirmed 4 years later from those used for the training dataset.

In this study, the pretrained LLM was fine-tuned with our training dataset. Pretraining of the LLM was conducted using Wikipedia data, which was devoid of radiological vocabularies. We found that fine-tuning of the LLM with radiological report data resulted in an improvement in its performance by increasing the number of training datasets from under 1000 to 1000 or more.

Class imbalance is known to be associated with an imbalanced sensitivity across each group [11]. Undersampling or oversampling are strategies to overcome this problem. Because it was relatively difficult to perform oversampling of data in groups other than group 2 (*n* = 2480), especially for group 0 (*n* = 37) and group 3 (*n* = 97) due to large difference in the number, we performed undersampling of group 2 data instead. The numbers of patients in groups 0/1/2/3 for the training dataset were 37/497/497/97 in session 5. Application of the undersampling technique resulted in an improved sensitivity for group 1 without compromising the sensitivity for group 2 in the validation dataset.

This study has some limitations. First, the number of patients in each group was lopsided. However, as it reflects real-world data, the proportion was not modified in the validation and test datasets. Conversely, undersampling of the largest group was conducted to balance the sensitivity across each group. Second, we used Bidirectional Encoder Representations from the Transformers Japanese model as the LLM. Because the performance of the model would differ across different languages, our results would not necessarily be applicable to other languages. Third, although chatGPT is gaining interest most recently [8, 12, 13], a comparison of the performance of our fine-tuned LLM with that of chatGPT was not conducted. This was due to the privacy issues associated with chatGPT [9], which requires uploading patient data to the server via the Internet. Fourth, external validation was not performed. However, we made efforts to enhance the generalizability of our study result (a) by avoiding overlap in radiologist who confirmed radiological report for the clinical purpose between training *vs.* validation and test datasets and (b) by setting 4 years interval for data collection between the training *vs.* validation and test datasets. Fifth, showing the benefit in the application of LLM to classification of patients on pretreatment for lung cancer with CT reports (clinical issue) was the main purpose of our study, thus, we did not intend to develop LLMs with state-of-the-art structure in this study. However, the fine-tuned model showed clinically feasible performance. Finally, the number of patients included in the training dataset was relatively small (*n* = 3111). However, our data revealed that the accuracy of the model reached a plateau when the number of patients in the training dataset exceeded 1000. Thus, the number of patients in the training dataset would be considered sufficient.

In conclusion, the fine-tuned LLM was effective in classifying patients on pretreatment for lung cancer based on radiological reports extracted from PACSs, with its performance comparable to that of radiologists in a remarkably shorter time.

## Data Availability

The datasets generated and/or analyzed during the current study are not publicly available due to patients' confidentiality.

## Abbreviations

AUC: Area under the receiver operating characteristic curve
LLM: Large language model
PACS: Picture archiving and communication systems
